# Postoperative Hypoxemia Caused by Atelectasis Due to an Unrecognized Tracheal Bronchus After Endoscopic Sinus Surgery: A Case Report

**DOI:** 10.7759/cureus.101387

**Published:** 2026-01-12

**Authors:** Mirei Sato, Eiki Kanemaru, Yohei Sakai, Shinya Suganuma, Takahisa Goto

**Affiliations:** 1 Department of Anesthesiology and Critical Care Medicine, Yokohama City University Hospital, Yokohama, JPN; 2 Department of Anesthesiology and Critical Care Medicine, Yokohama City University School of Medicine, Yokohama, JPN

**Keywords:** atelectasis, congenital heart disease, postoperative hypoxemia, rapid response team, tracheal bronchus

## Abstract

Tracheal bronchus is a rare airway anomaly prevalent in individuals with congenital heart disease (CHD). It is typically asymptomatic and diagnosed incidentally due to hypoxemia during general anesthesia in the operating room, where emergency procedures can be performed promptly. To the best of our knowledge, cases in which a tracheal bronchus induced severe hypoxemia after a patient’s transfer to the general ward have not been reported. Herein, we present a case in which a 46-year-old man with tuberous sclerosis and a history of ventricular septal defect (VSD) repair developed postoperative hypoxemia at the general ward after endoscopic sinus surgery. Bronchoscopy after reintubation revealed a tracheal bronchus originating approximately 2 cm above the carina that was occluded by aspirated blood, causing right upper lobe atelectasis. Bronchoscopic suction followed by lung recruitment improved oxygenation. This case highlights the importance of considering lobar atelectasis due to tracheal bronchial obstruction in the differential diagnosis of postoperative hypoxemia, particularly in patients with CHD.

## Introduction

Tracheal bronchus is a rare congenital airway anomaly with an incidence of 0.3%-1.0% [1] but occurs more frequently among individuals with congenital heart disease (CHD) [2]. Considering the increasing number of noncardiac surgeries performed in adults with CHD [3], it is essential to keep in mind the high prevalence of tracheal bronchus in this population. Tracheal bronchus is typically asymptomatic and diagnosed incidentally, commonly during general anesthesia, when the tracheal tube obstructs or is displaced into the anomalous bronchus, leading to atelectasis and hypoxemia [4-8]. To the best of our knowledge, cases of a tracheal bronchus causing postoperative hypoxemia after a patient’s transfer to the general ward have not been reported. Herein, we report a diagnostically challenging case of tracheal bronchus that caused hypoxemia requiring reintubation after endoscopic sinus surgery in a patient with a history of ventricular septal defect (VSD) repair.

## Case presentation

**Figure 1:**
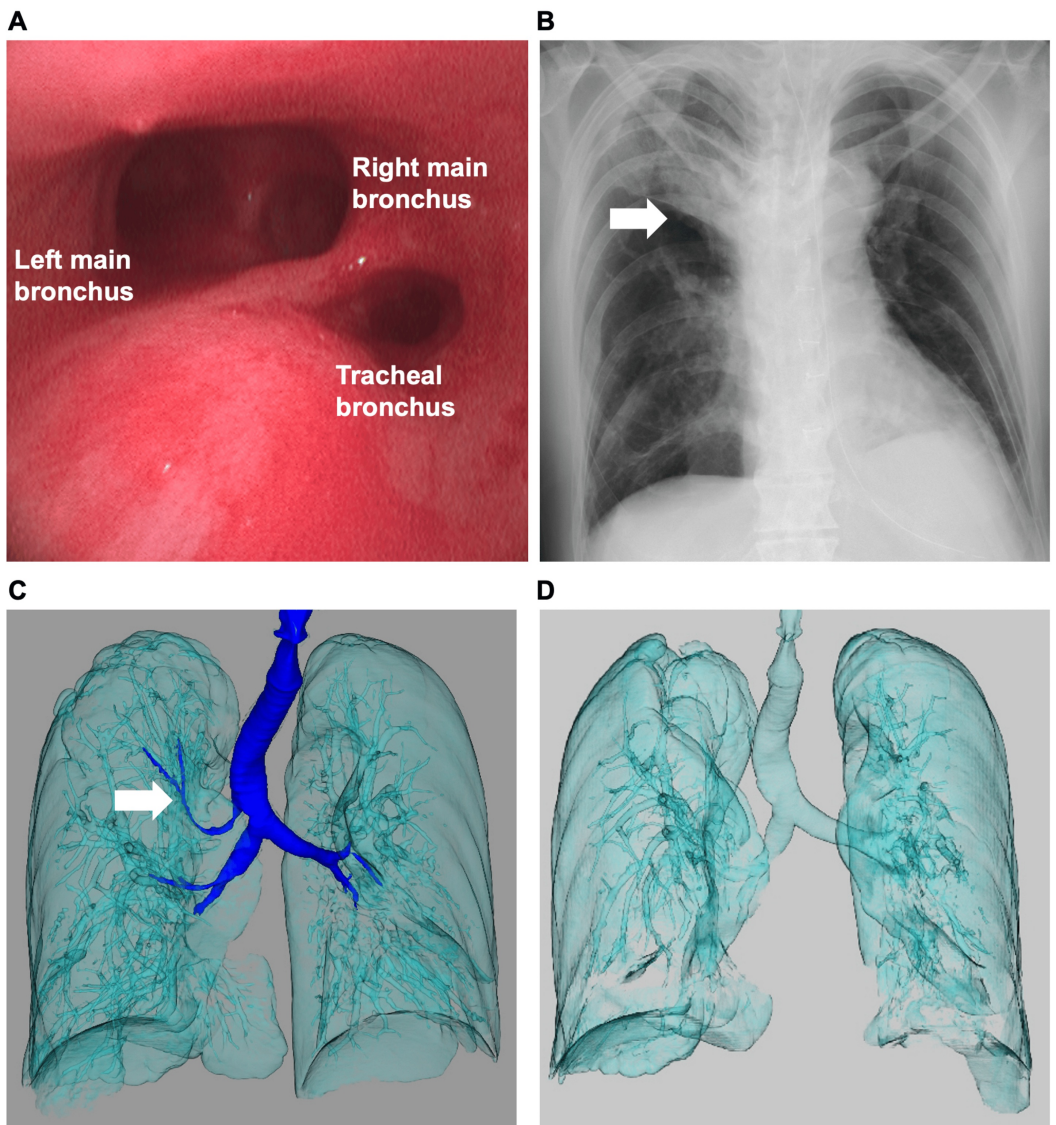
Bronchoscopic and radiological findings after reintubation A) Bronchoscopy image showing the orifice of a tracheal bronchus approximately 2 cm above the carina. B) Chest radiograph showing right upper lobe atelectasis (arrow) consistent with the territory of the tracheal bronchus. C) Three-dimensional computed tomography image showing a tracheal bronchus (arrow). D) Three-dimensional computed tomography image showing atelectasis in the right upper lobe, whereas the tracheal bronchus is no longer visualized due to obstruction by aspirated blood

The patient was a 46-year-old man with tuberous sclerosis complicated by epilepsy, intellectual disability, dysphagia, and a history of VSD repair. He underwent endoscopic sinus surgery for a paranasal sinus tumor under general anesthesia. The intraoperative course was uneventful. The patient was extubated in the operating room and transferred to the general ward. However, after the oxygen supply was discontinued one hour after arrival at the general ward, the peripheral oxygen saturation dropped to 80%. Consequently, a rapid response team was summoned to address the patient’s symptoms. Upon their arrival, peripheral oxygen saturation was 85% under 10 L/min of oxygen via a reservoir mask, and tachypnea was observed. Blood pressure was 116/74 mmHg, and heart rate was 94 beats/minute. Arterial blood gas analysis revealed hypoxemia and hypercapnia (pH, 7.3; PaO₂, 65.5 mmHg; PaCO₂, 70.8 mmHg; and lactate, 2.4 mmol/L). The differential diagnosis for the cause of postoperative hypoxemia included atelectasis, pulmonary thromboembolism, opioid-induced respiratory depression, and residual neuromuscular blockade. Since lung recruitment maneuvers did not improve oxygenation, atelectasis was ruled out. Transthoracic echocardiography showed no evidence of right heart enlargement or leftward displacement of the interventricular septum suggestive of pulmonary thromboembolism. Naloxone (0.2 mg) and sugammadex (200 mg) were administered for suspected residual opioid or neuromuscular blockade effects; however, the patient's condition did not improve. Due to persisting hypoxemia, the patient was admitted to the intensive care unit and reintubated. Bronchoscopy revealed a tracheal bronchus originating approximately 2 cm above the carina. Its orifice was obstructed by pooled blood (Figure 1A). Postintubation chest radiography showed right upper lobe atelectasis, which was consistent with the territory of the tracheal bronchus (Figure 1B). Three-dimensional computed tomography (CT) revealed that the tracheal bronchus, which was clearly visualized when the patient was healthy (Figure 1C), could no longer be visualized after obstruction by aspirated blood, and a noninflated/atelectatic lung was observed in the right upper lobe region (Figure 1D).

Bronchoscopic suction was performed to remove as much pooled blood as possible, followed by lung recruitment maneuvers to improve atelectasis and oxygenation. The patient received antibiotic therapy with ampicillin/sulbactam for aspiration pneumonia. He was extubated on postoperative day 5 and discharged on postoperative day 15.

## Discussion

This case highlights an unusual mechanism of postoperative hypoxemia. After being transferred to the general ward following endoscopic sinus surgery, the patient developed hypoxemia requiring a rapid response team intervention. Bronchoscopy after reintubation revealed that aspirated blood pooled in an unrecognized tracheal bronchus and occluded its orifice, resulting in right upper lobe atelectasis and hypoxemia. In contrast to previously reported cases of tracheal bronchi causing hypoxemia intraoperatively, in the present case, hypoxemia developed postoperatively at the general ward (Table 1).

**Table 1:** Case reports of perioperative hypoxemia in adult patients with a tracheal bronchus

Timing of hypoxemia	Place of occurrence of hypoxemia	Factor triggering hypoxemia	Reference
Postoperative	General ward	Tracheal bronchial orifice occlusion by aspirated blood	This case
From intraoperative to early post-extubation period	Operating room	Tracheal bronchial orifice occlusion by a tracheal tube	Tan et al. [5], Pribble et al. [6], Critchley et al. [7]
				Intraoperative	Operating room	Tracheal tube displacement into the tracheal bronchus	Sripada et al. [8]
				Intraoperative	Operating room	Tracheal bronchial orifice occlusion by a double-lumen tube	Iwamoto et al. [4]

Notably, our patient had a history of VSD repair, which is consistent with the reported association between tracheal bronchus and CHD. Given the increase in noncardiac surgeries performed in adults with CHD, lobar atelectasis caused by tracheal bronchial obstruction should be included in the differential diagnosis of unexplained postoperative hypoxemia in this population.

In patients with tracheal bronchus, general anesthesia is associated with a greater risk of hypoxia or atelectasis, making preoperative identification crucial. Nonetheless, this anomaly is rarely diagnosed before surgery because it is usually asymptomatic and not readily visible on preoperative imaging. While careful observation of preoperative chest radiographs can help identify a tracheal bronchus, its shadow may overlap with the mediastinal shadow, hindering its identification. Although multidetector CT is the gold standard for diagnosing tracheal bronchi [1], it is not performed unless tracheal bronchi are suspected preoperatively. This anomaly is usually diagnosed by fiberoptic bronchoscopy as an incidental finding during an unanticipated airway emergency under general anesthesia. The present case demonstrated that a tracheal bronchus can cause hypoxemia not only in the operating room but also in the general ward postoperatively, potentially leading to an airway emergency (Table 1).

Most tracheal bronchi originate less than 2 cm above the carina, albeit some have been identified as high as 6 cm above the carina [1,6]. Thus, in most patients, the tracheal tube does not interfere with the orifice of the tracheal bronchus, and surgeries under general anesthesia can be completed uneventfully. In our case, the tracheal bronchus orifice was located approximately 2 cm above the carina, and the tracheal tube was placed at a depth of 22 cm at the left corner of the mouth. Therefore, the tracheal tube did not obstruct the orifice or cause hypoxemia, which is why the tracheal bronchus was not detected during surgery.

The reported morbidity associated with a tracheal bronchus ranges from 0.3% in patients without CHD to 3.7%-12.8% in patients with CHD [2]. Furthermore, 43.7% of patients with a tracheal bronchus also have CHD [1]. The association between tracheal anomalies and CHD likely reflects shared embryologic origins, as both arise during overlapping stages of organogenesis. Accordingly, preoperative evaluation for a tracheal bronchus is warranted in patients with CHD.

The precise mechanism of aspiration in this case could not be determined with certainty. The patient also had tuberous sclerosis, a condition frequently accompanied by dysphagia. In addition, aspiration pneumonia is the most common cause of death in patients with tuberous sclerosis (10.5%) [9]. Postoperative bleeding is a significant complication of endoscopic sinus surgery, alongside cerebrospinal fluid leakage, meningitis, and orbital injury [10]. Taken together, persistent postoperative nasopharyngeal bleeding combined with the underlying dysphagia could have led to aspiration of blood in this case.

## Conclusions

We report a rare case in which an unrecognized tracheal bronchus caused severe postoperative hypoxemia after transfer to the general ward following endoscopic sinus surgery. Aspirated blood pooled within the tracheal bronchus and obstructed its orifice, resulting in right upper lobe atelectasis and respiratory failure requiring reintubation. This case expands previous reports by demonstrating that a tracheal bronchus can cause hypoxemia not only intraoperatively but also postoperatively outside the operating room. This case highlights the need to consider lobar atelectasis caused by tracheal bronchial obstruction in the differential diagnosis of unexplained postoperative hypoxemia, particularly in patients with CHD.

## References

[1] Lam K, Hoang LV, Anh LV (2022). Tracheal bronchus and associated pathologies detected by multidetector-row computed tomography in the Vietnamese population. J Clin Imaging Sci.

[2] Chen SJ, Lee WJ, Wang JK (2003). Usefulness of three-dimensional electron beam computed tomography for evaluating tracheobronchial anomalies in children with congenital heart disease. Am J Cardiol.

[3] Friedrich AJ, Radke R, Orwat S (2026). Non-cardiac surgeries in adults with congenital heart disease-influence of complexity of disease and estimated risk of surgery on adverse events. Eur J Intern Med.

[4] Iwamoto T, Takasugi Y, Hiramatsu K, Koga Y, Konishi T, Kozuka K, Murakami T (2009). Three-dimensional CT image analysis of a tracheal bronchus in a patient undergoing cardiac surgery with one-lung ventilation. J Anesth.

[5] Tan D, Gupta S, Block M (2022). Intraoperative diagnosis of "bronchus suis", a variant of tracheal bronchus. Cureus.

[6] Pribble CG, Dean JM (1994). An unusual cause of intraoperative hypoxemia. J Clin Anesth.

[7] Critchley LA, Ho AM, Lee SY (2007). Right upper lobe collapse secondary to an anomalous bronchus after endotracheal intubation for routine surgery. Anaesth Intensive Care.

[8] Sripada R, Keys K, Eichholz KM (2008). Inability to ventilate the lungs of a patient with tracheal anomaly and in prone position. J Clin Anesth.

[9] Kristof AS, Zhi Li P, Major P, Landry JS (2015). Lymphangioleiomyomatosis and tuberous sclerosis complex in Quebec: prevalence and health-care utilization. Chest.

[10] Saxena A, Nekhendzy V (2020). Anesthetic considerations for functional endoscopic sinus surgery: a narrative review. J Head Neck Anesth.

